# PARP1 Regulates the Biogenesis and Activity of Telomerase Complex Through Modification of H/ACA-Proteins

**DOI:** 10.3389/fcell.2021.621134

**Published:** 2021-05-19

**Authors:** Nikita V. Savelyev, Nikita M. Shepelev, Olga I. Lavrik, Maria P. Rubtsova, Olga A. Dontsova

**Affiliations:** ^1^Department of Chemistry, Lomonosov Moscow State University, Moscow, Russia; ^2^A.N. Belozersky Institute of Physico-Chemical Biology, Lomonosov Moscow State University, Moscow, Russia; ^3^Center of Life Sciences, Skolkovo Institute of Science and Technology, Skolkovo, Russia; ^4^Institute of Chemical Biology and Fundamental Medicine, Siberian Branch, Russian Academy of Sciences, Novosibirsk, Russia; ^5^Department of Natural Sciences, Novosibirsk State University, Novosibirsk, Russia; ^6^Shemyakin-Ovchinnikov Institute of Bioorganic Chemistry, Russian Academy of Sciences, Moscow, Russia

**Keywords:** telomerase, biogenesis, telomere, poly(ADP-Ribose) polymerase 1, H/ACA-proteins, ribonucleorprotein complex

## Abstract

Poly(ADP-ribose) polymerase 1 (PARP1) is established as a key regulator of the cellular DNA damage response and apoptosis. In addition, PARP1 participates in the global regulation of DNA repair, transcription, telomere maintenance, and inflammation response by modulating various DNA-protein and protein-protein interactions. Recently, it was reported that PARP1 also influences splicing and ribosomal RNA biogenesis. The H/ACA ribonucleoprotein complex is involved in a variety of cellular processes such as RNA maturation. It contains non-coding RNAs with specific H/ACA domains and four proteins: dyskerin (DKC1), GAR1, NHP2, and NOP10. Two of these proteins, DKC1 and GAR1, are targets of poly(ADP-ribosyl)ation catalyzed by PARP1. The H/ACA RNA-binding proteins are involved in the regulation of maturation and activity of the telomerase complex, which maintains telomere length. In this study, we demonstrated that of poly(ADP-ribosyl)ation influences on RNA-binding properties of DKC1 and GAR1 and telomerase assembly and activity. Our data provide the evidence that poly(ADP-ribosyl)ation regulates telomerase complex assembly and activity, in turn regulating telomere length that may be useful for design and development of anticancer therapeutic approaches that are based on the inhibition of PARP1 and telomerase activities.

## Introduction

Post-translational modifications regulate the localization, stability, and activity of proteins, thereby allowing the transformation of cellular signals into biological outcomes. PARP1 or poly(ADP-ribose) polymerase 1 catalyzes the covalent synthesis of the long branched polymer, poly(ADP-ribose) (PAR), utilizing nicotinamide adenine nucleotide (NAD+) as a substrate (Amé et al., 2004; Langelier et al., 2018). This reaction is reversible, as PAR is quickly hydrolyzed by poly(ADP-ribose) glycohydrolase (PARG) (Amé et al., 2004; Pascal and Ellenberger, 2015). PARG is an abundant enzyme that degrades PAR by a combination of endo- and exo-glycohydrolase activity, removing most of the PAR polymer but leaving behind a single ADP-ribose attached to the protein. This remnant ADP-ribosyl modification can be removed by mono-(ADP-ribose) glycohydrolases (Oka et al., 2006; Han et al., 2011; Kato et al., 2011). PARP1 is well established as a key regulator of the cellular DNA damage responses and the apoptotic machinery, but its functions are not restricted to only these processes (Hassa et al., 2006; Gagné et al., 2008). PARP1 functions in the global regulation of transcription (Frizzell et al., 2009), telomere maintenance (Tong et al., 2001; Espejel et al., 2004; Blasco, 2005; Beneke et al., 2008; Hoang et al., 2020), and inflammatory response (Francis et al., 1983; Bai et al., 2009).

Poly(ADP-ribosyl)ation (PARylation) regulates protein-protein and protein-nucleic acid interactions. The attachment of the large negatively charged ADP-ribose to the protein modulates the activity and interactome of the targeted protein. The influence of PARylation on protein-DNA interactions has been confirmed in different studies (Pinnola et al., 2007; Krishnakumar et al., 2008; Petesch and Lis, 2012a,b). PARylation has an influence on chromatin remodeling (Smeenk et al., 2013) and nucleosome-nucleosome interactions (Gomez et al., 2006; Pinnola et al., 2007; Schmutz et al., 2017), and many other processes. Recently, it was shown that PARP1 is involved in RNA biogenesis (Matveeva et al., 2019b). PARylation inhibits the activity of poly(A) polymerase (PAP) (Di Giammartino et al., 2013). PARP1 participates in the regulation of splicing (Matveeva et al., 2019a) and translation by modulating the activity of the heterogeneous nuclear ribonucleoproteins (hnRNPs) and mRNA-binding proteins (Kiss et al., 2010; Massenet et al., 2017; Melikishvili et al., 2017; Eleazer and Fondufe-Mittendorf, 2020; Hoang et al., 2020).

H/ACA RNA-protein complexes are involved in ribosome biogenesis, pre-mRNA splicing as well as in the assembly and stabilization of the human telomerase complex (Kiss et al., 2010; Massenet et al., 2017). The H/ACA ribonucleoprotein complex contains four proteins: DKC1, GAR1, NHP2, and NOP10, two of which (DKC1 and GAR1) have been identified as targets of PARP1 (Kiss et al., 2010; Zhang et al., 2013; Massenet et al., 2017). PARylation affects DNA-protein binding through different mechanisms: upon being PARylated, some proteins exhibit decreased affinity for DNA, while others exhibit increased affinity. This modulation is essential for various cellular processes and for the formation of chromatin structures (Kim, 2005). We investigated the influence of PARylation on the affinity of RNA-binding proteins to RNAs. We hypothesized that PARylation is also involved in the regulation of ribonucleoprotein complex assembly, structure, and function. To test this hypothesis, we analyzed the influence of PARylation of either GAR1 or DKC1 on the formation of ribonucleoprotein complexes. We analyzed the pattern of H/ACA RNA association with these proteins under different PARylation status, as well as the influence of PARylation on the activity and stability of the telomerase complex and telomere length and structure.

Telomerase is a key component of telomere maintaining system that is reactivated in majority of cancer cells as well as in cells with increased proliferation rate (Weng, 2008; Akincilar et al., 2016). Regulation of telomeres is important for cell survival, and is involved in healthy cell function, cell proliferation, aging and diseases such as cancer. An intimate understanding of the process of telomere lengthening and shortening at the molecular level is important in understanding of diverse cellular functions for long-term survival, disease prevention and reduce aging. The length of telomeres is dependent on the efficiency of telomerase assembly, stability, and activity. In this paper, the role of PARylation in these processes is described.

## Materials and Methods

### Cell Lines

HEK 293T and A549 cells were obtained from the ATCC and cultivated in DMEM-F12 containing 10% FBS and 1% penicillin/streptomycin in a humidified incubator at 37°C and 5% CO_2_. Cells in culture were treated with 50 μM olaparib for 3 h. To obtain lentiviral particles, the cells were transiently transfected with vectors and lentiviral plasmids using the calcium phosphate method. Lentiviral particles were harvested and used for HEK293T cell infection according to a previously published protocol (Weber et al., 2008). The cells were analyzed using an EVOS FL Imaging System (Thermo Fisher Scientific) and sorted using a FACSAria^TM^ III cell sorter (Bekton Dickinson). The cells were tested for mycoplasma contamination, which was confirmed to be negative.

### Plasmids and Transfection

The shPARP1 plasmid was constructed using the lentiviral gene ontology vector LeGO-Cer (Weber et al., 2008). The following oligonucleotides were used:

5′-ACCGAGGAAGGTATCAACAAATTTTCAAGAGAT TTGTTGATACCTTCCTCC-3′ (forward) and
5′-TCGAGGAGGAAGGTATCAACAAATCTCTTGAAA ATTTGTTGATACCTTCCTCGGT-3′ (reverse).

pcDNA3.1-3x FLAG-GAR1 was a kind gift from Egan and Collins (2010), and pMGIB-3x FLAG-DKC1 was a kind gift from S. Artandi (Addgene plasmid #53607) (Venteicher et al., 2009). 3x FLAG-DKC1 was cloned into the pcDNA3.1 vector under the same conditions for each experiment. The plasmids coding for the nonPARylated DKC1 and GAR1 were obtained through site-specific mutagenesis using PCR and Q5 High-Fidelity DNA Polymerase (NEB). Cells were either electroporated (1150 V, 2 × 20 ms impulses, Neon Transfection System, Thermo Fisher Scientific) or transfected with Lipofectamine 3000 (Thermo Fisher Scientific).

### Immunoblotting and Antibodies

For immunoblotting, cells were washed with PBS and lysed with ice-cold RIPA buffer (150 mM NaCl, 1% Triton X-100, 0.5% sodium deoxycholate, 0.1% SDS, 50 mM Tris–HCl pH 8.0) for 30 min with gentle agitation, and the protein concentration was determined using the Bradford method. The samples were denatured with 6xHU buffer + DTT (200 mM Tris–HCl pH 6.8, 10% glycerol, 5% SDS, 8 M urea, 1 mM EDTA, and 0.1% bromophenol blue), loaded onto gels and resolved at 180 V. The proteins were then transferred to PVDF membranes using a Bio-Rad Trans Blot SD system, blocked with 3% BSA/TBST, incubated with primary antibodies at 1/2000 dilution, washed four times in TBST, incubated with secondary antibodies at 1/5000 dilution, washed again, and developed using an ECL kit (GE Healthcare).

The following antibodies were used: TRF1 ab10579 (Abcam), TRF2 ab13579 (Abcam), PARP1 ab137653 (Abcam), TERT ab32020 (Abcam), DKC1 ab64667 (Abcam), β-actin ab8229 (Abcam), GAR1 11711-1-AP (Proteintech), PAR 4336-BPC (Trevigen), GAPDH 39-8600 (Thermo Fisher Scientific), α-tubulin ab18251 (Abcam), horseradish peroxidase-conjugated donkey anti-mouse A16011 (Thermo Fisher Scientific), and horseradish peroxidase-conjugated donkey anti-rabbit A16023 (Thermo Fisher Scientific).

### Telomere Length Analysis

For telomere Southern blotting, all procedures were performed as described previously (Liu, 2011). Genomic DNA was extracted from 2 million cells, digested with *Rsa*I and *Hinf*I (Thermo Fisher), resolved overnight at 30 V, transferred to Nytran SPC nitrocellulose membranes (Whatman) in 2x SSC, UV-crosslinked, and hybridized with ^32^P-labeled telomeric probes. Then, the membrane was washed, exposed to a ^32^P-sensitive cassette (GE) for several days, and imaged using a Typhoon FLA 9500 (GE).

### Telomerase Activity Assay

A TRAP assay was performed as described previously (Kim et al., 1994). For RQ-TRAP analysis, cells were counted, pelleted, washed with PBS, and lysed in TRAP lysis buffer (10 mM Tris–HCl, pH 7.5, 1 mM MgCl_2_, 0.1 mM PMSF, 5 mM β-mercaptoethanol, 1 mM EGTA, 5% glycerol, and 0.5% CHAPS). The cell extracts were diluted with 1x TRAP buffer (20 mM Tris-HCl pH 8.3, 1.5 mM MgCl_2_, 63 mM KCl, 1 mM EGTA, 0.1 mg/ml BSA, and 0.005% TWEEN 20) to equal cell equivalents. The diluted extract was incubated with TRAP mix 1 (TRAP 1x, dNTPs, and 100 ng of TS primer) at 2000 cell equivalents at 25°C for 30 min. TRAP mix 2 (H_2_O, Taq DNA polymerase, 100 ng of ACX primer, and SYBR Green I) was added, and qPCR was performed using a Bio-Rad CFX96/C1000 with an initial denaturation at 95°C for 2 min followed by 37 cycles of 95°C for 35 s, 50°C for 35 s, and 72°C for 90 s.

### Northern Blotting and qPCR

Northern blotting was performed as described previously (Xi and Cech, 2014; Shukla et al., 2016). Total RNA was extracted using the PureLink RNA Mini Kit (Thermo Fisher Scientific), and 20 μg RNA was resolved through electrophoresis on 5% polyacrylamide gels containing 7 M urea. Semidry transfer was used to transfer the resolved proteins to Nytran SPC nitrocellulose membranes (Whatman) at 400 mA in 1x TBE. The samples were UV-crosslinked and hybridized with ^32^P-labeled hTR or 7SL probes at 40°C. The samples were washed at 48°C, exposed to a ^32^P-sensitive cassette (GE), and imaged using a Typhoon FLA 9500 (GE).

RNA was also used for RT-qPCR analysis. The RNA was treated with DNase I (Thermo Fisher Scientific) and then cDNA was synthesized using a Maxima first strand cDNA synthesis kit (Thermo Fisher Scientific). qPCR was performed with 2x qPCR master mix (Thermo Fisher Scientific) on a Bio-Rad CFX96/C1000 with an initial denaturation of 95°C for 3 min followed by 30 cycles of 95°C for 30 s, 60°C for 30 s, and 72°C for 30 s.

### Telomerase Assembly Analysis

The telomerase assembly was analyzed as described previously (Azhibek et al., 2014). Cells were counted and lysed with TRAP lysis buffer, placed on top of a sucrose gradient in 1x TRAP buffer, and ultracentrifuged at 111132 × *g* at 4°C in a Beckman J2-HS. The fractions were collected starting from the top. Then, telomerase activity was measured as described in section “Telomerase Activity Assay.” For quantitating hTR, total RNA was extracted from each of the fractions with three volumes of TRIzol reagent (Thermo Fisher Scientific) according to the manufacturer’s protocol. As a control, an *in vitro*-synthesized hTR template was used. cDNA synthesis and qPCR were performed as described in section “Northern Blotting and qPCR.”

### Telomerase Precipitation and Immunoprecipitation

Cells were grown in three 175 cm^2^ flasks, detached using trypsin-EDTA, and counted. Equal amounts of cells were pelleted, washed with PBS, and lysed using telomerase buffer B (50 mM HEPES–KOH pH 8.0, 300 mM KCl, 2 mM MgCl_2_, 0.1% Triton X-100, 10% glycerol, 1 mM PMSF, 1 mM DTT, 0.15% CHAPS, and 1/100 volume Ribolock RI) for 30 min at 4°C (Azhibek et al., 2014). The lysates were centrifuged at 16000 × *g* at 4°C for 20 min, clarified for 1.5 h using protein G-Sepharose (Bialexa) blocked with 0.1% BSA, placed on top of 1 mL of telomerase buffer B with 20% glycerol, and centrifuged at 100000 × *g* for 45 min at 4°C. The supernatant was centrifuged again with 1 mL of telomerase buffer B with 20% glycerol at 210000 × *g* for 2.5 h at 4°C. Telomerase was dissolved in 1 mL of telomerase buffer B, incubated for 16 h at 4°C with hTERT antibodies, and immunoprecipitated with protein G-Sepharose for 6 h at 4°C. Sepharose was centrifuged at 2500 × *g* for 2 min and washed three times with telomerase buffer B. RNA and protein were eluted with TRIzol and extracted according to the manufacturer’s protocol.

cDNA was prepared as described in section “Northern Blotting and qPCR.” To determine the enrichment of the eluates with the target RNA, equal amounts of input and eluate RNA were used for cDNA synthesis, and qPCR was performed. For each cell line, the signal from the eluate was normalized to the signal from the input of the same sample. The ratio of enrichment between the cell lines was calculated.

For immunoprecipitation, 35 million cells were transfected with 60 μg of plasmid. After incubation for 2 days, the cells were harvested, washed with PBS, and lysed in IP buffer (50 mM HEPES-KOH pH 7.5, 150 mM KCl, 2 mM MgCl_2_, 0.1% Nonidet P-40, 10% glycerol, 1 mM EGTA, 1 mM PMSF, and 0.35 mM DTT) for 30 min at 4°C with rotation and then the cell lysate was centrifuged at 16000 × *g* at 4°C. M2 anti-FLAG agarose (Sigma) was washed with IP buffer and then added to the lysate. Immunoprecipitation was performed for 16 h at 4°C. The agarose was centrifuged at 2500 × *g* for 2 min and washed four times with IP buffer. RNA and protein were eluted with TRIzol and extracted according to the manufacturer’s protocol.

All experiments were performed a minimum of three times on different days.

For qPCR analysis, the standard deviations were calculated as described previously (Azhibek et al., 2014).

### Telomeric FISH

Telomeric FISH cells were synchronized in the G2/M phase using 100 ng/μl nocodazole (Sigma) treatment for 8 h. The cells were then treated with a hypotonic solution (0.075 M KCl, 8 g/L sodium citrate) and spreads of metaphase chromosomes with further FISH were produced as previously described (Ourliac-Garnier and Londoño-Vallejo, 2011).

All images were prepared using Nikon Ti2000 fluorescence microscope.

### Statistical Analysis

Data were analyzed by one- and two-way ANOVA and found significant (*p* < 0,05), and differences between the control and treated groups were determined using Šidák’s test with GraphPad Prism 8.0 software (La Jolla, CA, United States).

## Results

### PARylation Modulates the Affinity of H/ACA Proteins for RNAs

A global analysis of ADP-ribosylation revealed that the components of the H/ACA-complex (Zhang et al., 2013), DKC1 and GAR1, are targets of PARylation, and therefore, we decided to investigate the pattern of H/ACA RNA binding to PARylated and nonPARylated mutant forms of these proteins.

To clone mutant nonPARylated forms of DKC1 and GAR1, we performed site-directed mutagenesis at positions that were previously shown to be PARylated (Zhang et al., 2013): E414Q, E420Q, E429Q, E439Q, E483Q, and E487Q for DKC1; E67Q, E74Q, E80Q, D81N, and E104Q for GAR1.

**FIGURE 1:**
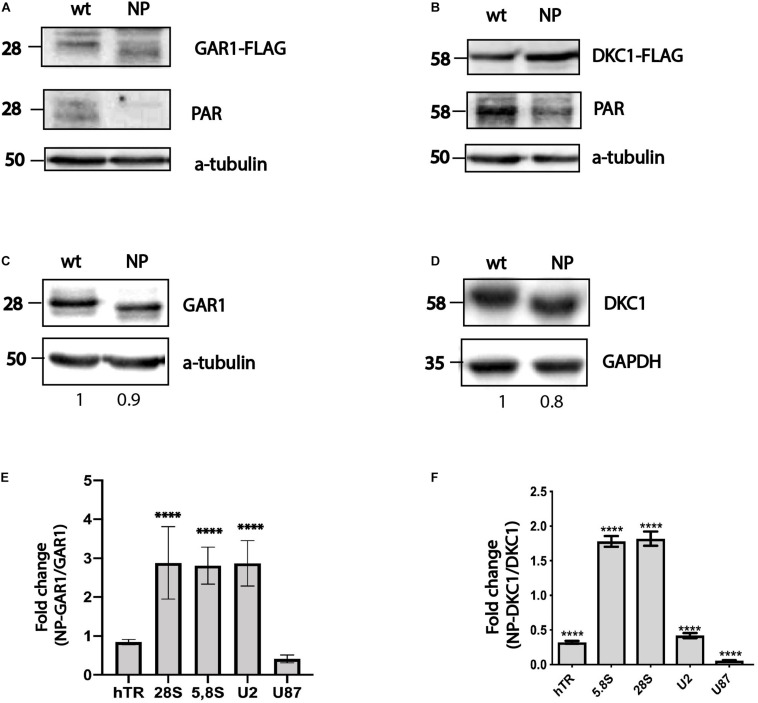
PARylation regulates ribonucleoprotein complex stability. Substitution of amino acid residues at sites proposed for PARylation influences the PAR-attachment to GAR1 **(A)** and DKC1 **(B)** mobility of the GAR1 **(A,C)** and DKC1 **(B,D)** proteins. Differential binding of RNA to GAR1 and NP-GAR1 **(E)** and to DKC1 and NP-DKC1 **(F)**, as revealed by RT-qPCR analysis. The mean values were calculated from triplicate RT-qPCR experiments with three biological replicates, and the bars represent SE. ^****^P < 0.0001 by Šidák’s multiple comparisons test.

To perform affinity purification of H/ACA RNA bound to either GAR1 or DKC1, constructs coding for both wild-type and mutant forms of 3xFLAG-DKC1 and 3xFLAG-GAR1 were used. We transfected the constructs into HEK293T cells and immunoprecipitated the expressed proteins using the 3xFLAG epitope through affinity purification. At first, we decided to check that introduced mutations disturb the PARylation performing the western blotting with the antibodies specific to FLAG-epitope and to PAR. We observed a shift in the bands corresponding to GAR1 and DKC1 in the wild-type and nonPARylated (NP) forms when the cellular extracts were analyzed through western blotting (Figures 1A–D) and the decreased interaction with PAR-specific antibodies (Figures 1A,B). We concluded that introduced mutations prevent the PARylation of GAR1 and DKC1 that results in the decreased molecular weight of proteins that was observed by western blotting (Figures 1C,D). RNA associated with the precipitated proteins was purified and used for RT-qPCR analysis. We chose a set of RNAs that are well known targets of DKC1 and GAR1, including ribosomal RNAs (5,8S, and 28S), telomerase RNA (hTR), and a number of snRNAs, for the analysis. To compare the levels of co-purified RNAs, we calculated the yield of RNA after immunoprecipitation by normalizing the amount of a particular RNA in the eluate to the amount of the same RNA in the input fraction. To compare the results for nonPARylated mutants with those for wild-type proteins, the RNA yields (described above) precipitated with nonPARylated proteins were normalized to the yields precipitated with the wild-type proteins. We observed that PARylation could increase or decrease the binding of a particular H/ACA RNA with DKC1 and GAR1 (Figures 1E,F). We observed accumulation of ribosomal RNAs and decreased amounts of snRNAs and hTR complexed with NP DKC1 (Figures 1E,F) and accumulation of ribosomal RNA and U2 snRNA complexed with NP GAR1 in comparison with wild-type proteins. The level of hTR bound with NP GAR1 was not changed and slight reduction of U87 bound with NP GAR1 in comparison with wild type GAR1 was observed. These results demonstrated the existence of different modes of regulation of the ribonucleoprotein complex biogenesis by PARylation.

**FIGURE 2:**
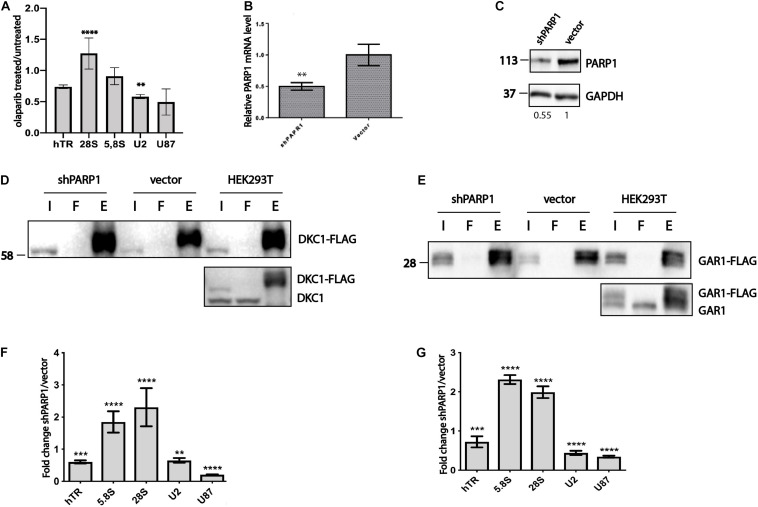
PARylation influences the ability of H/ACA proteins to bind to RNA. **(A)** RT-qPCR analysis of the levels of RNA co-immunoprecipitated with GAR1 after olaparib treatment and without treatment. The level of RNA associated with GAR1 in cells treated with olaparib was normalized to the level of RNA associated with GAR1 in cells without treatment. The mean values were calculated from triplicate RT-qPCR experiments with three biological replicates, and the bars represent SE. ^****^P < 0.0001 and ^∗∗^*P* < 0.01 using Šidák’s multiple comparisons test. **(B)** The expression of a shRNA targeting PARP1 mRNA inhibits PARP1 expression, as revealed by RT-qPCR. ** indicates unpaired *t*-test two tailed *p* value < 0,01. **(C)** PARP1 levels are decreased in cells expressing shRNA targeting PARP1 mRNA, as revealed by western blotting. **(D)** Immunoprecipitation of DKC1-3xFLAG from HEK293T cells was followed by immunoblotting with anti-FLAG antibodies (upper panel) and with anti-DKC1 antibodies (lower panel). I – input, F – flow-through, E – elution. **(E)** Immunoprecipitation of GAR1-3xFLAG from HEK293T cells was followed by immunoblotting with anti-FLAG antibodies (upper panel) and anti-GAR1 antibodies (lower panel). I – input, F – flow-through, E – elution. **(F)** RT-qPCR analysis of the levels of RNA co-immunoprecipitated with DKC1. The mean values were calculated from triplicate RT-qPCR experiments with three biological replicates, and the bars represent SE. ^****^P < 0.0001 and ^∗∗∗^*P* < 0.001 using Šidák’s multiple comparisons test. **(G)** RT-qPCR analysis of the levels of RNA co-immunoprecipitated with GAR1. The mean values were calculated from triplicate RT-qPCR experiments with three biological replicates, and the bars represent SE. ^****^P < 0.0001, ^∗∗∗^*P* < 0.001, and ^∗∗^*P* < 0.01 using Šidák’s multiple comparisons test.

The nonPARylated forms of GAR1 and DKC1 were obtained by mutagenesis; we changed six amino acid residues in DKC1 and five in GAR1. Mutations in proteins can change their tertiary structures, resulting in changes in the protein function independent of the modification status. To confirm that the absence of PARylation, rather than altered physicochemical properties of the mutant proteins, influenced their affinity for RNA, we analyzed the RNA-binding properties of DKC1 and GAR1 when the activity of PARPs was inhibited. We treated cells with the olaparib known inhibitor of the PARPs that is used as a therapeutic substance for the medical treatment of cancer. Cells exogenously expressed wild-type form of 3xFLAG-GAR1 were incubated for 3 h with the 50 μM of olaparib. Cellular extracts were obtained and used for immunoprecipitation. RNA associated with the precipitated proteins was purified and used for RT-qPCR analysis. To compare the levels of co-purified RNAs, we calculated the yield of RNA after immunoprecipitation by normalizing the amount of a particular RNA in the eluate to the amount of the same RNA in the input fraction. To compare the results the RNA yields (described above) precipitated with GAR1 from treated with olaparib cells were normalized to the yields precipitated with GAR1 from untreated cells (Figure 2A). We observed that hTR binding with GAR1 was decreased while the level of 28S ribosomal RNA associated with the GAR1 increased in cells treated with olaparib. These data partially confirm the results obtained when we used the nonPARylated mutant form of GAR1 (Figure 1E) and reinforce the conclusion that the PARylation regulates the RNA-binding ability of H/ACA-proteins.

The effect of PARP1 on the RNA-binding property of H/ACA proteins was confirmed additionally in cells when the expression of PARP1 was decreased by RNA interference. We generated a stable cell line with decreased expression levels of PARP1 by transducing HEK293T cells with lentivirus containing the LeGo-Cer (Weber et al., 2008) construct. This construct encodes an shRNA specific to PARP1 mRNA, as described previously (Wu et al., 2013), as well as a Cer fluorescent protein that enables sorting of the infected cells. A cell line stably expressing the empty LeGo-Cer vector was used as a control. The obtained cell line was characterized using a senescence-associated β-galactosidase test (Lee et al., 2006), because the senescence phenotype has been previously observed in cells with decreased levels of PARP1 expression (Lee et al., 2006; Wang et al., 2014). We treated the cells with 100 nM doxorubicin for 4 days to induce senescence and measured the β-galactosidase activity. We observed increased number of cells with the senescence phenotype in the shPARP1 cell line compared to the control cell line expressing the empty vector (Supplementary Figure 1). The level of PARP1 mRNA decreased 2-fold (Figure 2B) and the level of PARP1 protein decreased 1.8-fold (Figure 2C) in the knockdown cells compared to that in the control cells, as demonstrated by RT-qPCR and western blotting, respectively.

The obtained cell lines were transfected with DKC1-3xFLAG and GAR1-3xFLAG constructs and then subjected to co-immunoprecipitation of DKC1 and GAR1 (Figures 2D,E). We performed co-immunoprecipitation, purified RNA from the eluted fractions, and analyzed the copurified RNAs by RT-qPCR. The efficiency of immunoprecipitations was controlled by western blotting analysis of input, flow-through and elution fractions using anti-FLAG antibodies (Figures 2D,E, upper panel). The level of exogenous and endogenous DKC1 and GAR1 were found comparable by western blotting analysis of fractions from HEK293T cells with antibodies specific to DKC1 (Figure 2D, lower panel) or GAR1 (Figure 2E, lower panel). To compare the obtained data, the RNA levels in the eluted fractions were normalized to the levels of RNA in the input fractions. The calculated levels of copurified RNA from cells with decreased levels of PARP1 were also normalized to those of copurified RNA from control cells expressing empty vector. We observed that the amount of ribosomal RNA associated with DKC1 (Figure 2F) and GAR1 (Figure 2G) increased, while the amounts of hTR and H/ACA-RNA decreased in PARP1-deficient cells compared to control cells. We observed a very similar effect of PARylation on the pattern of RNA binding with DKC1 and GAR1, regardless of the approach used for inhibition of PARylation, either through site-directed mutagenesis of modified amino acid residues or total inhibition of PARP1 expression by RNA interference.

**FIGURE 3:**
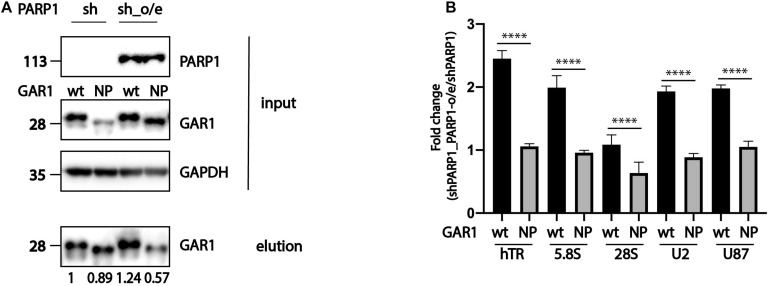
PARylation modulates the ability of RNA-binding proteins to associate with RNA. **(A)** Western blot analysis of expression of PARP1, GAR1, and NP-GAR1 (input panel) and efficiency of immunoprecipitation (elution panel). **(B)** RT-qPCR analysis of the levels of RNA co-immunoprecipitated with GAR1. The mean values were calculated from triplicate RT-qPCR experiments with three biological replicates, and the bars represent SE. ^****^P < 0.0001.

To demonstrate the direct influence of PARP1 activity on the ability of RNA-binding proteins to associate with RNA, we overexpressed wild-type PARP1 and various forms of GAR1 (wild type and mutant nonPARylated form) in cells expressing shPARP1, which have decreased levels of PARP1. We decided to concentrate our study on RNAs associated with GAR1 because previous experiment (Figures 1A,B) demonstrated that mutations completely prevent the PARylation of GAR1 but not DKC1. Cells with decreased levels of PARP1 (shPARP1) were transfected with GAR1-3xFLAG and GAR1-NP-3xFLAG, and PARP1 construct and subjected to co-immunoprecipitation with GAR1 protein. We performed immunoprecipitation, purified RNA from the eluted fractions, and analyzed the copurified RNAs by RT-qPCR. The expression of PARP1, GAR1, and immunoprecipitation quality was confirmed through western blotting (Figure 3A). The RNA levels in the eluted fractions were normalized to the RNA levels in the input fractions. The calculated levels of copurified RNA from cells overexpressing wild-type GAR1 or NP-GAR1 with overexpression of PARP1 were normalized to the levels of copurified RNA from cells with decreased levels of PARP1. The relative levels of proteins eluted during immunoprecipitation were used as coefficients in the calculation of RNA associated with GAR1 protein. The obtained data are presented in Figure 3B. We observed that the overexpression of PARP1 increased the level of RNA associated with wild-type GAR1 up to two times, while there were no differences in the association of RNA with the nonPARylated form of GAR1 (Figure 3B).

### PARP1 Is Involved in Human Telomerase Complex Biogenesis and Stability

**FIGURE 4:**
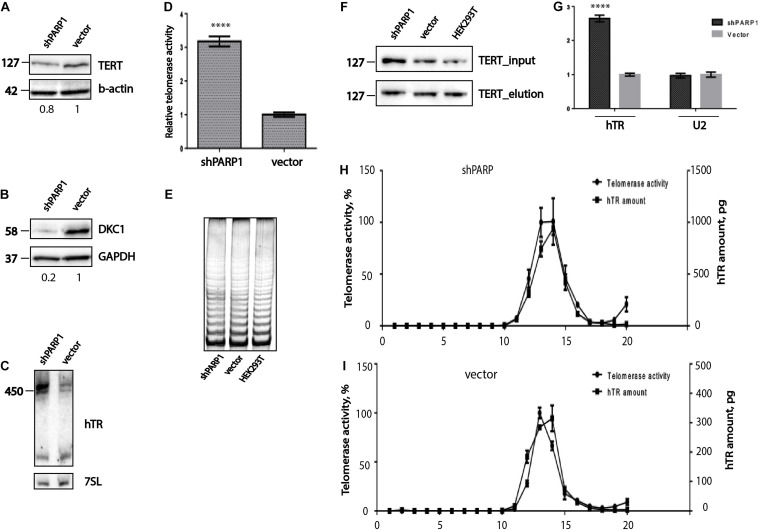
PARP1 is involved in the regulation of telomerase complex composition and stability. **(A)** Western blot analysis of TERT protein levels in the indicated cell lines. **(B)** Western blot analysis of DKC1 protein levels in the indicated cell lines. **(C)** Northern blot analysis of hTR expression in the indicated cell lines. **(D)** RQ-TRAP telomerase activity analysis in the indicated cell lines. ^****^indicates unpaired *t*-test two tailed *p* value < 0,0001. **(E)** Analysis of telomerase processivity using TRAP assay followed by PAGE separation of PCR products. **(F)** Immunoprecipitation of hTERT from the indicated cell lines was followed by immunoblotting with anti-hTERT antibodies. **(G)** RT-qPCR analysis of the amounts of hTR co-immunoprecipitated with hTERT. U2 RNA was used as a control. ^****^indicates unpaired *t*-test two tailed *p* value < 0,0001. **(H)** Analysis of hTR distribution and telomerase activity after separation of extracts from HEK293T cells expressing a shRNA targeting PARP1 mRNA, using a sucrose gradient. **(I)** Analysis of hTR distribution and telomerase activity after separation of extract from HEK293T cells expressing the empty LeGo-Cer vector, using a sucrose gradient.

PARylation is involved in the regulation of telomere maintenance in mammalian cells (Espejel et al., 2004; Beneke et al., 2008). Inhibition of PARP1 expression by RNA interference using siRNA leads to rapid shortening of telomeres, but does not influence telomerase activity (Beneke et al., 2008). PARP1 regulates the binding of TRF2, resulting in telomere shortening (Gomez et al., 2006). We used a stable cell line that constitutively expresses shPARP1, specific to the PARP1 mRNA, to investigate the regulation of telomere maintenance. We determined the levels of telomerase compounds and the efficiency of telomerase complex association in cells that were PARP1 deficient. Interestingly, the levels of hTERT (Figure 4A) and DKC1 (Figure 4B) decreased in cells with PARP1 knockdown. However, the level of hTR increased (Figure 4C) after inhibition of PARP1 gene expression. We observed an accumulation of the mature full-length form of hTR, while the levels of the previously observed fragments of the degraded hTR (Li and Blackburn, 2006; Figure 4C) was unchanged. Cells with decreased levels of PARP1 demonstrated increased levels of telomerase activity, as revealed by RQ-TRAP analysis (Figure 4D), however, the processivity of telomerase was not affected (Figure 4E), as observed by TRAP followed by electrophoresis. In order to verify the general effect of long-term inhibition of expression of PARP1 on telomerase activity, we generated A549 cells stably producing short hairpin specifically inhibited PARP1 expression as well as an empty vector. Obtained cell lines were used for the analysis of PARP1 mRNA level and telomerase activity (Supplementary Figure 2). Unfortunately, the rate of inhibition of PARP1 expression was not dramatic. We observed that level of PARP1 mRNA decreased by 30% (Supplementary Figure 2A) and statistically unreliable increasing of telomerase activity (Supplementary Figure 2B). To improve the influence of PARP1 inhibition on telomerase activity we additionally treated cells with olaparib and observed a slight increase of telomerase activity. These data provide additional evidence of the general effect of PARP1 influence on telomerase function in the cell.

We proposed that decreased level of PARP1 influences on telomerase assembly. We co-immunoprecipitated the telomerase complex from HEK293T cells, cells expressing the LeGo-Cer vector, and shPARP1-expressing cells, using anti-hTERT antibodies. It was demonstrated that comparable levels of hTERT were purified from the different cell lines (Figure 4F). Quantitative analysis of the copurified hTR was performed with the help of RT-qPCR. The results demonstrated that the levels of hTR were 3-fold higher in cells expressing shPARP1 than in cells expressing the LeGo-Cer vector (Figure 4G).

To confirm the effects on telomerase complex assembly, we obtained extracts from cells with PARP1 knockdown and from control cells expressing empty vector and separated them using sucrose gradient centrifugation (Figures 4H,I; Azhibek et al., 2014). In the control cells as well as in the cells with PARP1 knockdown, we observed that the peak hTR levels correlated with the peak telomerase activity. Three times higher amount of telomerase RNA was associated with the active telomerase in cells with decreased level of PARP1 compared to that in the control cells (Figures 4H,I).

**FIGURE 5:**
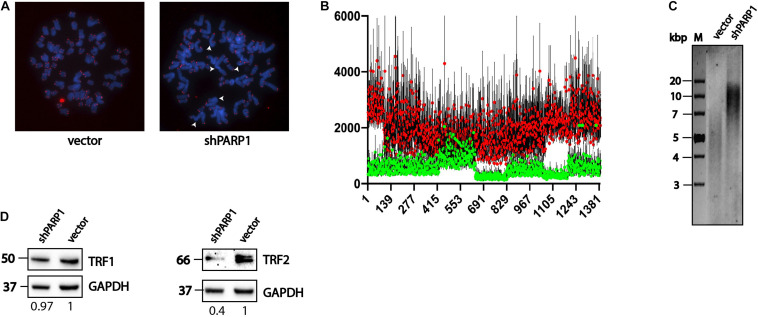
PARP1 is involved in the regulation of telomere length and telomerase activity. **(A)** Immunofluorescence-FISH analysis of metaphase spreads from cells expressing the LeGo-Cer vector and a shRNA specific to PARP1 mRNA. White arrows point defects in telomeric structures. **(B)** Telomere length analysis performed by Telometer software by the Johns Hopkins University. Green dots correspond control cells (vector) and red dots correspond spreads from cells with decreased level of PARP1 (shPARP1). **(C)** Telomere restriction fragment length analysis of PARP1 knockdown cells. **(D)** Western blot analysis of TRF1 protein levels in the indicated cell lines. **(E)** Western blot analysis of TRF2 protein levels in the indicated cell lines.

We decided to analyze the influence of increased stability and activity of the telomerase complex on telomere structure. We observed increased numbers of fused chromosomes (Figure 5A) and increased telomere length (Figure 5B) using FISH. The fused chromosome number increased from 3% in wild type to 12% in cells with a decreased level of PARP1. Increased telomere length was confirmed by Southern blotting (Figure 5C). The level of TRF1 protein was unaffected (Figure 5D), however, the level of TRF2 decreased significantly in the shPARP1 cell line compared to that in the control cell line (Figure 5E).

## Discussion

Poly(ADP-ribose) polymerase 1 is involved in the cellular response to DNA damage (Tong et al., 2001). Telomeres are special DNA-protein structures that protect the ends of the linear eukaryotic chromosomes from the DNA damage recognition system (de Lange, 2018). The involvement of PARP1 in the maintenance of telomere length has been previously demonstrated (Gomez et al., 2006; Beneke et al., 2008; Schmutz et al., 2017; de Lange, 2018). PARP1 interacts with the telomeres through the TRF2 protein (Gomez et al., 2006; Schmutz et al., 2017). PARP1 accumulates at critically short telomeres (Gomez et al., 2006) and protects them from being exposed to DNA-damaging reagents. PARP1 participates in t-loop cleavage by recruiting HJ-resolvases (Schmutz et al., 2017; de Lange, 2018). Short-term inhibition of PARP1 in HeLa cells by treatment with siRNAs or unspecific chemicals results in telomere shortening (Beneke et al., 2008). However, there are reports that such treatment slightly inhibits tankyrases but does not affect telomere length (Wahlberg et al., 2012; de Lange, 2018). PARP1-null mouse cells do not demonstrate telomere dysfunction (Espejel et al., 2004). Therefore, data on the influence of PARylation on telomere length are very controversial. We have demonstrated that long-term inhibition of PARP1 in HEK293T cells, using stable expression of shRNA specific to PARP1 mRNA, results in telomere lengthening (Figure 5B) and accumulation of different telomere defects, such as telomere signal-free ends and chromosome and chromatid end-to-end fusions (Figure 5A). The same effects have been demonstrated previously in cells with inhibited activity of PARP1 due to exposure to DNA-damaging treatments (Gomez et al., 2006). Telomere restriction fragment analysis in cells with decreased expression of PARP1 after a long period of culturing (more than 50 passages) revealed the presence of elongated telomeres (Figure 5B). Unexpectedly, telomerase activity was increased in these cells (Figure 4D). Telomerase activity decreased in response to short-term inhibition of PARP1 expression, in contrast to the effects of long-term PARP1 inhibition. However, in agreement with the data for short-term inhibition, we also found decreased levels of TRF2 in PARP1-deficient cells (Figure 5D), which may lead to telomeres being more accessible to telomerase. The level of hTERT decreased (Figure 4A), which correlates with the previous observations of inhibition of hTERT transcription mediated by the KLF4 transcription factor (Hsieh et al., 2017). The levels of DKC1 and hTERT (Figures 4A,B) decreased in cells with PARP1 knockdown that may have resulted in decreased telomerase activity. However, in the same cells, the levels of hTR increased (Figure 4C), which may have resulted as a stabilization response of the telomerase complex in the absence of PARylation. To assess this possibility, we analyzed telomerase complex assembly by co-immunoprecipitation with hTERT (Figures 4F,G) and centrifugation in a sucrose concentration gradient (Azhibek et al., 2014; Figures 4H,I). In all cases, hTR was present in the active telomerase complex, and its concentration increased 3-fold in PARP1-deficient cells compared to control cells. We observed that PARylation influences the binding of a particular RNA (Figures 1E,F, 2F,G, 3B) by H/ACA proteins such as DKC1 and GAR1. Taken together, our results demonstrate that PARylation of DKC1 and GAR1 regulates the RNA-binding properties of these proteins in the hTR complex.

In this study, we clearly demonstrated that long-term inhibition of PARP1 expression increased telomere length and telomerase activity. Increase in the levels of total hTR and hTR associated with the telomerase complex resulted in activation of the telomerase enzyme and elongation of telomeres. Decrease in the levels of TRF2 allowed telomerase to associate with telomeres more effectively, which may have resulted in telomere elongation.

These observations should be taken into consideration to enhance the understanding, design, and development of anticancer therapeutic approaches that are based on the inhibition of PARP1 and telomerase activities. It should be mentioned that the inhibition of these important cellular compounds, involved in nuclear genome stability, might have various effects on cell survival mechanisms.

## Data Availability Statement

The original contributions presented in the study are included in the article/Supplementary Material, further inquiries can be directed to the corresponding author/s.

## Author Contributions

NSa and NSh performed the experiments. MR contributed to the design and analysis of all experiments, conceived and supervised the project, and wrote the manuscript. OL contributed to the design and discussion of experimental data and wrote the manuscript. OD supervised the project and wrote the final version of the manuscript. All authors contributed to the article and approved the submitted version.

## Conflict of Interest

The authors declare that the research was conducted in the absence of any commercial or financial relationships that could be construed as a potential conflict of interest.

## References

[B1] Akincilar S. C. Unal B. Tergaonkar V. (2016). Reactivation of telomerase in cancer. *Cell. Mol. Life Sci.* 73 1659–1670. 10.1007/s00018-016-2146-9 26846696 PMC4805692

[B2] Amé J.-C. Spenlehauer C. de Murcia G. (2004). The PARP superfamily: review articles. *BioEssays* 26 882–893. 10.1002/bies.20085 15273990

[B3] Azhibek D. Zvereva M. Zatsepin T. Rubtsova M. Dontsova O. (2014). Chimeric bifunctional oligonucleotides as a novel tool to invade telomerase assembly. *Nucleic Acids Res.* 42 9531–9542. 10.1093/nar/gku688 25081209 PMC4150790

[B4] Bai P. Hegedűs C. Szabó É Gyüre L. Bakondi E. Brunyánszki A. (2009). Poly(ADP-Ribose) polymerase mediates inflammation in a mouse model of contact hypersensitivity. *J. Invest. Dermatol.* 129 234–238. 10.1038/jid.2008.196 18633442

[B5] Beneke S. Cohausz O. Malanga M. Boukamp P. Althaus F. Bürkle A. (2008). Rapid regulation of telomere length is mediated by poly(ADP-ribose) polymerase-1. *Nucleic Acids Res.* 36 6309–6317. 10.1093/nar/gkn615 18835851 PMC2577345

[B6] Blasco M. A. (2005). Telomeres and human disease: ageing, cancer and beyond. *Nat. Rev. Genet.* 6 611–622. 10.1038/nrg1656 16136653

[B7] de Lange T. (2018). Shelterin-mediated telomere protection. *Annu. Rev. Genet.* 52 223–247. 10.1146/annurev-genet-032918-021921 30208292

[B8] Di Giammartino D. C. Shi Y. Manley J. L. (2013). PARP1 Represses PAP and inhibits polyadenylation during heat shock. *Mol. Cell* 49 7–17. 10.1016/j.molcel.2012.11.005 23219533 PMC3545032

[B9] Egan E. D. Collins K. (2010). Specificity and stoichiometry of subunit interactions in the human telomerase holoenzyme assembled in vivo. *Mol. Cell. Biol.* 30 2775–2786. 10.1128/MCB.00151-10 20351177 PMC2876521

[B10] Eleazer R. Fondufe-Mittendorf Y. N. (2020). The multifaceted role of PARP1 in RNA biogenesis. *Wiley Interdiscip. Rev. RNA* 12:e12607. 10.1002/wrna.1617 32656996 PMC7856298

[B11] Espejel S. Klatt P. Murcia J. M. Martín-Caballero J. Flores J. M. Taccioli G. (2004). Impact of telomerase ablation on organismal viability, aging, and tumorigenesis in mice lacking the DNA repair proteins PARP-1, Ku86, or DNA-PKcs. *J. Cell Biol.* 167 627–638. 10.1083/jcb.200407178 15545322 PMC2172587

[B12] Francis G. E. Gray D. A. Berney J. J. Wing M. A. Guimaraes J. E. Hoffbrand A. V. (1983). Role of ADP-ribosyl transferase in differentiation of human granulocyte-macrophage progenitors to the macrophage lineage. *Blood* 62 1055–1062. 6313096

[B13] Frizzell K. M. Gamble M. J. Berrocal J. G. Zhang T. Krishnakumar R. Cen Y. (2009). Global analysis of transcriptional regulation by poly(ADP-ribose) polymerase-1 and poly(ADP-ribose) glycohydrolase in MCF-7 human breast cancer cells. *J. Biol. Chem.* 284 33926–33938. 10.1074/jbc.M109.023879 19812418 PMC2797163

[B14] Gagné J.-P. Isabelle M. Lo K. S. Bourassa S. Hendzel M. J. Dawson V. L. (2008). Proteome-wide identification of poly(ADP-ribose) binding proteins and poly(ADP-ribose)-associated protein complexes. *Nucleic Acids Res.* 36 6959–6976. 10.1093/nar/gkn771 18981049 PMC2602769

[B15] Gomez M. Wu J. Schreiber V. Dunlap J. Dantzer F. Wang Y. (2006). PARP1 Is a TRF2-associated poly(ADP-Ribose)polymerase and protects eroded telomeres. *Mol. Biol. Cell* 17 1686–1696. 10.1091/mbc.e05-07-0672 16436506 PMC1415310

[B16] Han W. Li X. Fu X. (2011). The macro domain protein family: structure, functions, and their potential therapeutic implications. *Mutat. Res.* 727 86–103. 10.1016/j.mrrev.2011.03.001 21421074 PMC7110529

[B17] Hassa P. O. Haenni S. S. Elser M. Hottiger M. O. (2006). Nuclear ADP-ribosylation reactions in mammalian cells: where are we today and where are we going? *Microbiol. Mol. Biol. Rev.* 70 789–829. 10.1128/MMBR.00040-05 16959969 PMC1594587

[B18] Hoang S. M. Kaminski N. Bhargava R. Barroso-González J. Lynskey M. L. García-Expósito L. (2020). Regulation of ALT-associated homology-directed repair by polyADP-ribosylation. *Nat. Struct. Mol. Biol.* 27 1152–1164. 10.1038/s41594-020-0512-7 33046907 PMC7809635

[B19] Hsieh M.-H. Chen Y.-T. Chen Y.-T. Lee Y.-H. Lu J. Chien C.-L. (2017). PARP1 controls KLF4-mediated telomerase expression in stem cells and cancer cells. *Nucleic Acids Res.* 45 10492–10503. 10.1093/nar/gkx683 28985359 PMC5737510

[B20] Kato J. Zhu J. Liu C. Stylianou M. Hoffmann V. Lizak M. J. (2011). ADP-ribosylarginine hydrolase regulates cell proliferation and tumorigenesis. *Cancer Res.* 71 5327–5335. 10.1158/0008-5472.CAN-10-0733 21697277 PMC3399181

[B21] Kim M. Y. (2005). Poly(ADP-ribosyl)ation by PARP-1: ‘PAR-laying’ NAD+ into a nuclear signal. *Genes Dev.* 19 1951–1967. 10.1101/gad.1331805 16140981

[B22] Kim N. W. Piatyszek M. A. Prowse K. R. Harley C. B. West M. D. Ho P. L. (1994). Specific association of human telomerase activity with immortal cells and cancer. *Science* 266 2011–2015. 7605428 10.1126/science.7605428

[B23] Kiss T. Fayet-Lebaron E. Jády B. E. (2010). Box H/ACA small ribonucleoproteins. *Mol. Cell* 37 597–606. 10.1016/j.molcel.2010.01.032 20227365

[B24] Krishnakumar R. Gamble M. J. Frizzell K. M. Berrocal J. G. Kininis M. Kraus W. L. (2008). Reciprocal binding of PARP-1 and histone H1 at promoters specifies transcriptional outcomes. *Science* 319 819–821. 10.1126/science.1149250 18258916

[B25] Langelier M.-F. Eisemann T. Riccio A. A. Pascal J. M. (2018). PARP family enzymes: regulation and catalysis of the poly(ADP-ribose) posttranslational modification. *Curr. Opin. Struct. Biol.* 53 187–198. 10.1016/j.sbi.2018.11.002 30481609 PMC6687463

[B26] Lee B. Y. Han J. A. Im J. S. Morrone A. Johung K. Goodwin E. C. (2006). Senescence-associated β-galactosidase is lysosomal β-galactosidase. *Aging Cell* 5 187–195. 10.1111/j.1474-9726.2006.00199.x 16626397

[B27] Li S. Blackburn E. H. (2006). Expression and suppression of human telomerase RNA. *Cold Spring Harb. Symp. Quant. Biol.* 71 211–215. 10.1101/sqb.2006.71.009 17381299

[B28] Liu D. (2011). “Analysis of average telomere length in cultured human cells,” in *Telomeres and Telomerase*, ed. Songyang Z. (Totowa, NJ: Humana Press), 13–19. 10.1007/978-1-61779-092-8_2 21461807

[B29] Massenet S. Bertrand E. Verheggen C. (2017). Assembly and trafficking of box C/D and H/ACA snoRNPs. *RNA Biol.* 14 680–692. 10.1080/15476286.2016.1243646 27715451 PMC5519232

[B30] Matveeva E. A. Al-Tinawi Q. M. H. Rouchka E. C. Fondufe-Mittendorf Y. N. (2019a). Coupling of PARP1-mediated chromatin structural changes to transcriptional RNA polymerase II elongation and cotranscriptional splicing. *Epigenetics Chromatin* 12:15. 10.1186/s13072-019-0261-1 30777121 PMC6378753

[B31] Matveeva E. A. Mathbout L. F. Fondufe-Mittendorf Y. N. (2019b). PARP1 is a versatile factor in the regulation of mRNA stability and decay. *Sci. Rep.* 9:3722. 10.1038/s41598-019-39969-7 30842529 PMC6403249

[B32] Melikishvili M. Chariker J. H. Rouchka E. C. Fondufe-Mittendorf Y. N. (2017). Transcriptome-wide identification of the RNA-binding landscape of the chromatin-associated protein PARP1 reveals functions in RNA biogenesis. *Cell Discov.* 3:17043. 10.1038/celldisc.2017.43 29387452 PMC5787697

[B33] Oka S. Kato J. Moss J. (2006). Identification and characterization of a mammalian 39-kDa poly(ADP-ribose) glycohydrolase. *J. Biol. Chem.* 281 705–713. 10.1074/jbc.M510290200 16278211

[B34] Ourliac-Garnier I. Londoño-Vallejo A. (2011). Telomere length analysis by quantitative fluorescent in situ hybridization (Q-FISH). *Methods Mol. Biol.* 735 21–31. 10.1007/978-1-61779-092-8_3 21461808

[B35] Pascal J. M. Ellenberger T. (2015). The rise and fall of poly(ADP-ribose): an enzymatic perspective. *DNA Repair* 32 10–16. 10.1016/j.dnarep.2015.04.008 25963443 PMC4522361

[B36] Petesch S. J. Lis J. T. (2012a). Activator-induced spread of poly(ADP-Ribose) polymerase promotes nucleosome loss at Hsp70. *Mol. Cell* 45 64–74. 10.1016/j.molcel.2011.11.015 22178397 PMC3473076

[B37] Petesch S. J. Lis J. T. (2012b). Overcoming the nucleosome barrier during transcript elongation. *Trends Genet.* 28 285–294. 10.1016/j.tig.2012.02.005 22465610 PMC3466053

[B38] Pinnola A. Naumova N. Shah M. Tulin A. V. (2007). Nucleosomal core histones mediate dynamic regulation of poly(ADP-ribose) Polymerase 1 protein binding to chromatin and induction of its enzymatic activity. *J. Biol. Chem.* 282 32511–32519. 10.1074/jbc.M705989200 17827147

[B39] Schmutz I. Timashev L. Xie W. Patel D. J. de Lange T. (2017). TRF2 binds branched DNA to safeguard telomere integrity. *Nat. Struct. Mol. Biol.* 24 734–742. 10.1038/nsmb.3451 28805810

[B40] Shukla S. Schmidt J. C. Goldfarb K. C. Cech T. R. Parker R. (2016). Inhibition of telomerase RNA decay rescues telomerase deficiency caused by dyskerin or PARN defects. *Nat. Struct. Mol. Biol.* 23 286–292. 26950371 10.1038/nsmb.3184 PMC4830462

[B41] Smeenk G. Wiegant W. W. Marteijn J. A. Luijsterburg M. S. Sroczynski N. Costelloe T. (2013). Poly(ADP-ribosyl)ation links the chromatin remodeler SMARCA5/SNF2H to RNF168-dependent DNA damage signaling. *J. Cell Sci.* 126 889–903. 10.1242/jcs.109413 23264744

[B42] Tong W.-M. Hande M. P. Lansdorp P. M. Wang Z.-Q. (2001). DNA strand break-sensing molecule poly(ADP-Ribose) polymerase cooperates with p53 in telomere function, chromosome stability, and tumor suppression. *Mol. Cell. Biol.* 21 4046–4054. 10.1128/MCB.21.12.4046-4054.2001 11359911 PMC87066

[B43] Venteicher A. S. Abreu E. B. Meng Z. McCann K. E. Terns R. M. Veenstra T. D. (2009). A human telomerase holoenzyme protein required for cajal body localization and telomere synthesis. *Science* 323, 644–648. 10.1126/science.1165357 19179534 PMC2728071

[B44] Wahlberg E. Karlberg T. Kouznetsova E. Markova N. Macchiarulo A. Thorsell A.-G. (2012). Family-wide chemical profiling and structural analysis of PARP and tankyrase inhibitors. *Nat. Biotechnol.* 30 283–288. 10.1038/nbt.2121 22343925

[B45] Wang H. Lu C. Tan Y. Xie J. Jiang J. (2014). Effect of adriamycin on BRCA1 and PARP-1 expression in MCF-7 breast cancer cells. *Int. J. Clin. Exp. Pathol.* 7 5909–5915. 25337234 PMC4203205

[B46] Weber K. Bartsch U. Stocking C. Fehse B. (2008). A multicolor panel of novel lentiviral “gene ontology” (LeGO) vectors for functional gene analysis. *Mol. Ther. J. Am. Soc. Gene Ther.* 16 698–706. 10.1038/mt.2008.6 18362927

[B47] Weng N. (2008). Telomere and adaptive immunity. *Mech. Ageing Dev.* 129 60–66. 10.1016/j.mad.2007.11.005 18199471 PMC2276146

[B48] Wu W. Kong Z. Duan X. Zhu H. Li S. Zeng S. (2013). Inhibition of PARP1 by small interfering RNA enhances docetaxel activity against human prostate cancer PC3 cells. *Biochem. Biophys. Res. Commun.* 442 127–132. 10.1016/j.bbrc.2013.11.027 24239883

[B49] Xi L. Cech T. R. (2014). Inventory of telomerase components in human cells reveals multiple subpopulations of hTR and hTERT. *Nucleic Acids Res.* 42 8565–8577. 10.1093/nar/gku560 24990373 PMC4117779

[B50] Zhang Y. Wang J. Ding M. Yu Y. (2013). Site-specific characterization of the Asp- and Glu-ADP-ribosylated proteome. *Nat. Methods* 10 981–984. 10.1038/nmeth.2603 23955771

