# Supplementary material for: PARP1 Regulates the Biogenesis and Activity of Telomerase Complex Through Modification of H/ACA-Proteins
Source: Front Cell Dev Biol. 2021 May 19;9:621134. doi: 10.3389/fcell.2021.621134 (PMC8170401; doi:10.3389/fcell.2021.621134)
Supplement: Supplementary file 1 [file Data_Sheet_1.docx]

**Senescence induction**

The assay was done according to (Lee et al., 2006). Cells were treated with 100 nM doxorubicin for 4 days and then fixed in 2% formaldehyde/0.2% glutaraldehyde in PBS for 5 min. Cells were washed thrice with PBS and incubated in β-galactosidase staining solution for 18 h at 37°C (40 mM citric acid/sodium phosphate buffer pH 6.0, 150 mM NaCl, 2 mM MgCl_2_, 5 mM K_3_Fe(CN)6, 5 mM K_4_Fe(CN)_6_, and 1 mg/mL X-Gal). The cells were washed again in PBS for 10 min, fixed for 5 min in methanol, and imaged using the Evos Cell Imaging System (Thermo Fisher Scientific).

**Supplementary Figure 1.** PARP1 is involved in the regulation of telomere length and the senescence phenotype. **(A)** Analysis of the senescence phenotype in the indicated cell lines by β-galactosidase activity assay. The dark colored cells are senescent. **(B)** qPCR analysis of telomere length in the indicated cell lines.


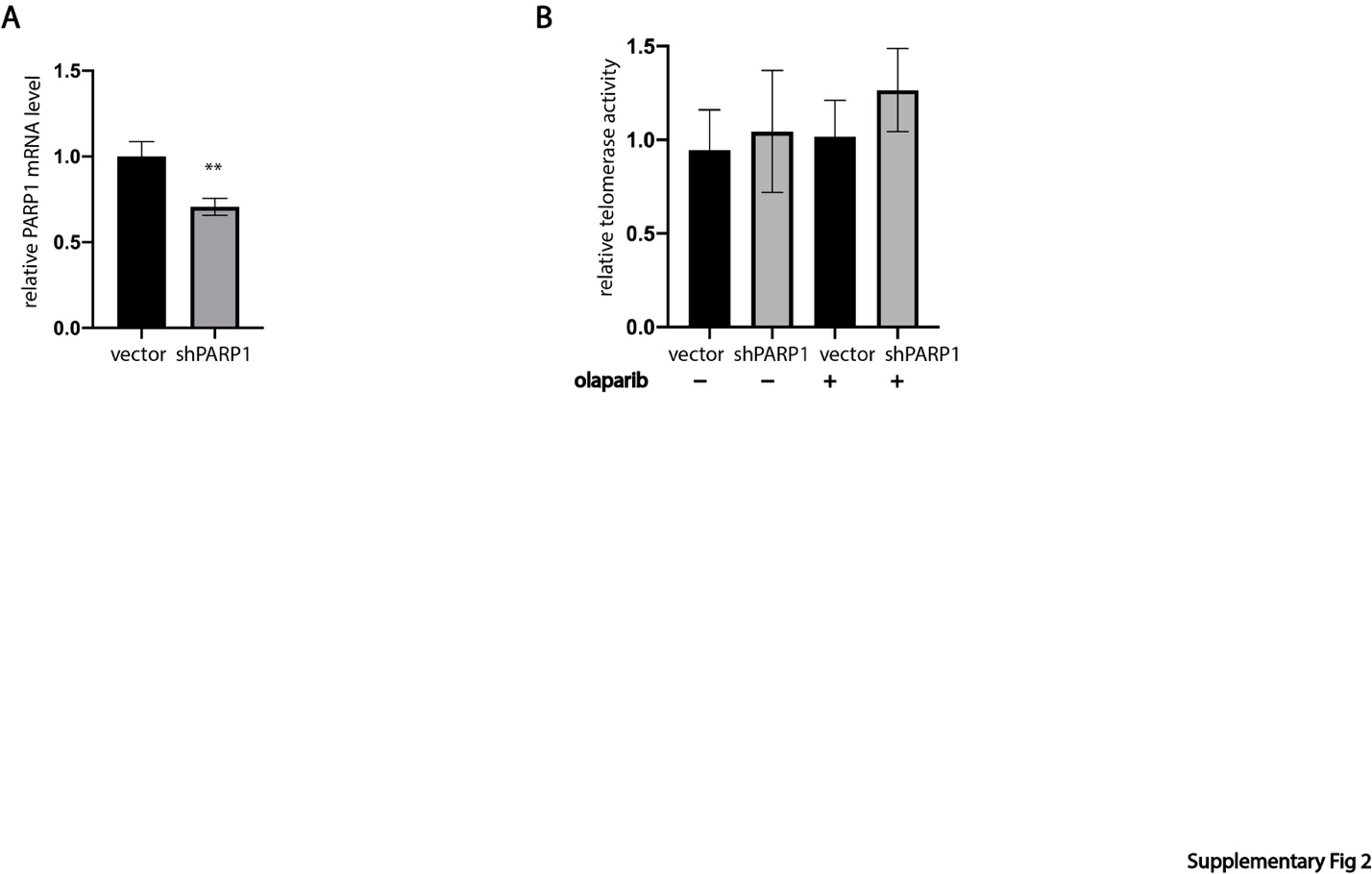


**Supplementary Figure 2.** PARP1 is involved in the regulation of telomerase activity in A549 cells. **(A)** The expression of a shRNA targeting PARP1 mRNA inhibits PARP1 expression, as revealed by RT-qPCR. ** indicates unpaired t-test two tailed p value<0,01. **(B)** RQ-TRAP telomerase activity analysis in the indicated cell lines. Telomerase activity was determined in untreated and treated with 50μM olaparib.
